# Role of the Aspartate Transaminase and Platelet Ratio Index in Assessing Hepatic Fibrosis and Liver Inflammation in Adolescent Patients with HBeAg-Positive Chronic Hepatitis B

**DOI:** 10.1155/2015/906026

**Published:** 2015-07-05

**Authors:** Yu Zhijian, Li Hui, Yao Weiming, Lin Zhanzhou, Chen Zhong, Zheng Jinxin, Wang Hongyan, Deng Xiangbin, Yang Weizhi, Li Duoyun, Liu Xiaojun, Deng Qiwen

**Affiliations:** ^1^Department of Infectious Diseases and Shenzhen Key Lab for Endogenous Infection, Shenzhen Nanshan Hospital, Guangdong Medical College, No. 89, Taoyuan Road, Nanshan District, Shenzhen 518052, China; ^2^Department of Gastroenterology, The Central Hospital of Wuhan, No. 26, Jianghan Road, Wuhan, China; ^3^Institute of Infectious Diseases and Medical Center for Liver Failure, Beijing 302 Hospital, Beijing, China; ^4^Department of Liver Diseases, Huizhou Municipal Central Hospital, No. 41 Eling North Road, Huizhou, China

## Abstract

This study described an index of aspartate aminotransferase-to-platelet ratio index (APRI) to assess hepatic fibrosis with limited expense and widespread availability compared to the liver biopsy in adolescent patients with CHB.

## 1. Introduction

Hepatitis B virus (HBV) infection is a major cause of chronic liver disease. Globally, there are over 400 million people infected with HBV, and infection-related complications such as severe liver failure have become a significant global health problem [1]. It is well known that the exact staging of liver fibrosis and accurate degree of liver inflammation are crucial for us to make the therapeutic decision and assess the prognosis of CHB patients [2]. However, till now, we still mainly depend on liver biopsies to assess their disease status, including the degree of liver fibrosis and grade of inflammation [1, 2]. Liver biopsies are an invasive examination with possible traumatic complications including bleeding, pain, and pneumothorax and, therefore, these methods cannot be frequently repeated and not suitable for dynamic surveillance of the pathological changes of hepatic tissues [3–5]. Recently, several noninvasive diagnostic models of liver fibrosis have been established using combinations of serological markers, such as the aspartate transaminase and platelet ratio index (APRI), Hepascore, Fibroindex, slower lung function growth (SLFG), Fibroscan, Fibrotouch, and Forn's index [6–18]. The APRI has the advantage of including only 2 inexpensive laboratory tests and easy performance routinely in all patients. Mounting evidences have shown the clinical value of the APRI in predicting liver fibrosis and inflammation with various etiologies and, especially worthy of our attention, the detection of this index has been recommended to evaluate and monitor the liver disease status in the WHO guideline of treatment and prevention of HBV [3]. However, there are few studies conducted to assess the APRI for predicting the fibrosis stage of adolescents with HBV-related fibrosis and inflammation; the objective of this study was to investigate the diagnostic accuracy of the APRI for the prediction of significant fibrosis and inflammation in this population.

## 2. Materials and Methods

### 2.1. Patient Enrollment

Eighty-eight HBeAg-positive patients who met the inclusion criteria were selected from our hospitals between July 2007 and July 2013. The patient population included 57 males and 31 females, with an average age of 8.9 ± 5.6 years (range: 1 to 18). The following inclusion criteria were applied: CHB diagnosis conforms to the relevant criteria based on the Viral Hepatitis Prevention and Treatment Programs as previously reported [2]; the patient was an adolescent (defined as being 1 to 18 years of age); and the patient had not received any antiviral therapy and any other treatment before liver biopsy. We investigated the radiographic evaluation (including ultrasonic examination, upper abdominal CT, or MRI) and analyzed clinical manifestations to exclude decompensated liver cirrhosis of these patients in this study. Patients with other types of hepatitis and liver cancer were excluded. The experimental scheme was approved by the Medical Ethics Committee of our hospital and patients or their parents signed an informed consent form.

### 2.2. Liver Biopsy

Hepatic function, routine blood tests, hepatitis B surface markers, and HBV DNA quantification were evaluated within 1 day before liver biopsy. Liver samples with the length of over 15 mm were taken twice from the region of interest by a Bard Magnum biopsy gun with diameter 1.4 mm. Two passages of hepatic tissue samples obtained from every patient were then sliced, stained, and observed under a microscope. The Scheuer scheme was adopted as the pathological diagnostic standard [3, 4, 15]. The degree and type of fibrosis were divided into five stages [3, 4, 15]: S0 (no fibrosis), S1 (periportal fibrosis without septa), S2 (portal fibrosis with few septa), S3 (numerous septa, bridging fibrosis), and S4 (cirrhosis). For analytical purposes, stages S2 or higher were considered indicative of significant fibrosis. The degree and types of inflammation were also divided into five stages [3, 4, 15]: G0 (no inflammation), G1 (portal inflammatory infiltration, slight spotty necrosis, or focal necrosis), G2 (portal or periportal inflammatory necrosis without piecemeal necrosis, spotty necrosis, focal necrosis, and acidophilic degeneration), G3 (interface hepatitis, confluent necrosis, and bridging necrosis), and G4 (widely bridging necrosis and piecemeal necrosis). For analytical purposes, stages G2 or higher were considered indicative of significant inflammation.

### 2.3. APRI Determination

An automatic biochemistry analyzer and an automated hematology analyzer measured the aspartate transaminase (AST) and platelet (PLT) levels just one day before the liver biopsy. The APRI was calculated as follows: AST (ULN)/PLT (109/L) [14].

### 2.4. Statistical Analysis

SPSS16.0 statistical software was used for statistical analysis. Quantitative data was expressed as *X* ± *S*, an independent-samples *t*-test was used for comparison between two groups, and an ANOVA was used for comparison between more than two groups. Qualitative information R × C table was expressed by *χ* ^2^ test. Spearman correlation analysis was used to inspect the degree of correlation between the APRI and liver fibrosis and inflammation. Logistic regression analysis was used for the prediction of risk indicator of liver fibrosis and inflammation. The result of pathological examination was taken as the “gold standard” to calculate sensitivity (Se) and specificity (Sp) of liver fibrosis and inflammation that were corresponding with each diagnostic cut-off value of the APRI and to make the receiver operating characteristic (ROC) curve. The area under the receiver operating characteristic curve (AUROC) was calculated by Hanley-McNeil nonparameter, calculating the corresponding positive predictive value (PPV) and negative predictive value (NPV). Differences were considered statistically significant at *P* < 0.05.

## 3. Results

### 3.1. The APRI Significantly Predicts Liver Fibrosis and Inflammation in Adolescent Patients with CHB

In our cohort of 88 adolescent patients with CHB, 45 cases had mild liver fibrosis (S1; 51.1%), and 43 had significant liver fibrosis (≥S2; 48.9%), including 11 cases with S3 fibrosis and one with S4 fibrosis. The APRI value (0.86 ± 0.58) for patients with mild liver fibrosis was significantly lower than the patients with significant fibrosis (1.39 ± 0.95; *P* < 0.01). The basic patient characteristics of the groups with mild and significant fibrosis are shown in Table S1 in Supplementary Material available online at http://dx.doi.org/10.1155/2015/906026. Logistic regression analysis of those indicators that were significantly different revealed that albumin (ALB) (*P* = 0.036), PLT (*P* = 0.044), and APRI (*P* = 0.005) were predictive indices of the risk of liver fibrosis, with an OR of 0.85 (95% CI: 0.73–0.99), 0.99 (95% CI: 0.98–1.00), and 2.85 (95% CI: 1.38–5.89), respectively.

With regard to liver inflammation, 31 patients had mild liver inflammation (G1; 35.2%), and 57 patients had significant liver inflammation (≥G2; 64.8%), including five cases with G3 inflammation and one with G4. The basic patient characteristics of the groups with mild and significant liver inflammation are shown in Table S2. Statistical analysis revealed that the APRI value (0.75 ± 0.46) in the patients with mild liver inflammation was significantly lower than the group with significant inflammation (1.32 ± 0.90; *P* < 0.01). Logistic regression analysis revealed that ALB (*P* = 0.010), cholinesterase (CHE) (*P* = 0.000), and APRI (*P* = 0.005) were predictive indices of the risk of liver inflammation, with an OR of 0.82 (95% CI: 0.70–0.95), 0.99 (95% CI: 0.99–1.00), and 4.16 (95% CI: 1.55–11.18), respectively.

**Figure 1:**
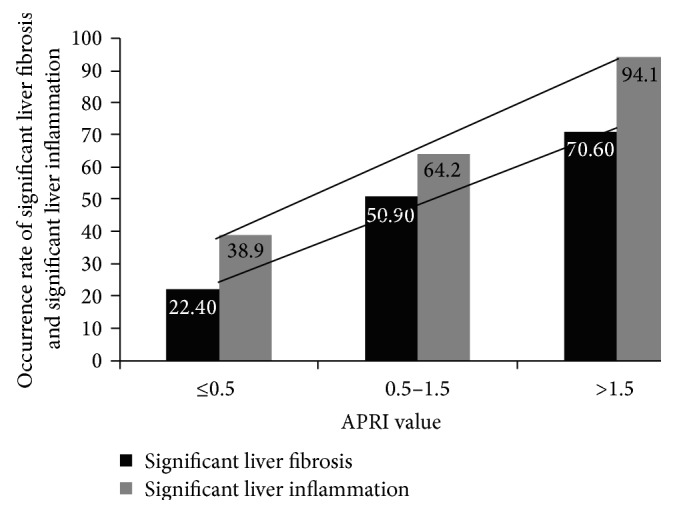
The percentage of significant liver fibrosis and significant liver inflammation in different groups with differential APRI values.

If an APRI value of 0.5 and 1.5 was taken as the cut-off value to divide patients into three groups (≤0.5, 0.5–1.5, and >1.5), the percentage of patients with significant liver fibrosis (≥S2) in each group was 22.2% (4/18), 51.9% (27/54), and 75% (12/16) and that with significant liver inflammation (≥G2) was 38.9% (7/18), 64.8% (35/54), and 94.1% (15/16), respectively. These values were significantly different (*P* < 0.05; Figure 1), suggesting liver fibrosis and liver inflammation gradually worsened with an increasing APRI value. Indeed, Spearman correlation analysis showed that the APRI was significantly correlated with liver fibrosis degrees (*rs* = 0.65, *P* < 0.01) and inflammation stages (*rs* = 0.47, *P* < 0.01), respectively.

### 3.2. Clinical Significance of Different APRI Cut-Off Values to Predict Liver Fibrosis and Inflammation

**Figure 2:**
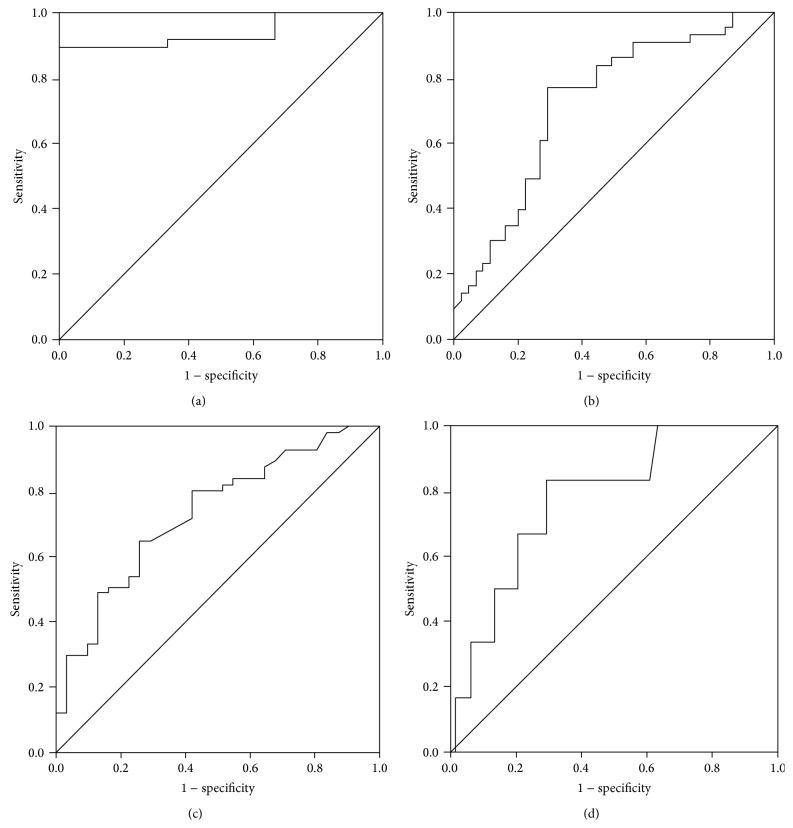
ROCs for fibrosis stages (≥S1 and ≥S2) were shown in (a) and (b), respectively, and for inflammation degrees (≥G2 and ≥G3) shown in (c) and (d), respectively, using APRI model.

ROC analysis of the APRI was used to predict the degree of liver fibrosis in our patient population. In those patients with ≥S1 stage liver fibrosis, the AUROC of the APRI was 0.94 (95% CI: 0.87–1.01, *P* < 0.05). Using an APRI of 0.40 as a cut-off point to judge patients who developed liver fibrosis (≥S1), the corresponding PPV and NPV were 98.7% and 18.2%, respectively (Figure 2(a) and Table 1). In patients with ≥S2 stage liver fibrosis, the AUROC of the APRI was 0.73 (95% CI: 0.62–0.83, *P* < 0.05). Using an APRI of 0.83 as a cut-off point to judge patients who developed significant liver fibrosis (≥S2), the corresponding PPV and NPV were 71.7% and 76.2% (Figure 2(b) and Table 1). Furthermore, when an APRI of 0.5 and 1.5 was taken as cut-off points, the corresponding PPV and NPV were 55.7%, 77.8% and 70.6%, 56.3%, respectively (Table 1). Importantly, a high APRI (cut-off ≥ 1.5) indicated a high specificity of significant liver fibrosis and inflammation.

ROC analysis of the APRI was also used to determine its predictive value with respect to liver inflammation. In those patients with ≥G2 stage inflammation stage, the AUROC of the APRI was 0.74 (95% CI: 0.63–0.84, *P* < 0.05). Using an APRI of 0.85 as a cut-off point to judge patients who developed significant liver inflammation (≥G2), the corresponding PPV and NPV were 82.2% and 53.5%, respectively (Figure 2(c) and Table 2). Moreover, when an APRI of 0.5 and 1.5 was taken as cut-off points, the corresponding PPV and NPV were 71.4%, 61.1% and 94.1%, 42.3%, respectively. In patients with ≥G3 inflammation, the AUROC of the APRI was 0.78 (95% CI: 0.61–0.95, *P* < 0.05) (Figure 2(d) and Table 2).

### 3.3. Predictive Values of APRI on Liver Fibrosis and Inflammation at Different Ages of Adolescent CHB Patients

In order to evaluate the correlation between age and liver fibrosis or inflammation using the APRI, we divided our patient population into three age groups, including those patients that were ≤6 years old, between 6 and 12 years old, and >12 years old. Based on the results described above, a cut-off value for the APRI to predict liver fibrosis and inflammation was set at 0.83 and 0.85, respectively. The diagnostic value of this index is shown in Tables 2 and 3. This analysis shows that, in patients between 6 and 12 years of age, the APRI have its advantages in predicting liver fibrosis and inflammation.

## 4. Discussion

The APRI may be the most accurate and simple marker for predicting significant fibrosis in CHB and CHC. Although clinical significance of this index in WHO guideline of prevention and treatment of HBV infection is recommended for assessing the degree of liver fibrosis [1–3], its predictive value is still needed to be demonstrated in the adolescents population with HBV-related fibrosis. In this study, we found that the APRI was significantly higher in adolescent patients with HBeAg-positive CHB who have significant fibrosis, versus those patients with mild fibrosis. We also found that the APRI indicated significant differences between adolescents with mild and significant inflammation, suggesting that the APRI may closely be correlated with liver disease progression, including the degree of fibrosis and the grade of inflammation. Our linear regression analysis also indicated the correlation between APRI and liver fibrosis/inflammation. Our data also indicated that when the APRI cut-off was set at 0.83, a comparatively rational sensitivity (76.7%) and specificity (71.1%) can be achieved to predict liver fibrosis stages.

With regard to the relationship between APRI and inflammation, several studies have shown that the APRI is associated with liver necroinflammation in the patients with CHB and CHC [6, 7]. However, few reports confirmed the clinical significance of the APRI in Chinese adolescent patients with CHB and, in this study, our data indicated a close correlation between APRI and liver inflammation in adolescent patients with CHB. Specifically, our data indicated that, with an APRI cut-off set at 0.85, it is possible to have a comparatively rational sensitivity (70.6%) and specificity (83.3%), especially in patients between 6 and 12 years old. Furthermore, when patients were stratified by age, our data showed that, in patients between 6 and 12 years of age, these cut-off values of 0.83 and 0.85 may have APRI advantages to predict significant fibrosis and significant inflammation, respectively. Our data is consistent with previous reports; for example, it was previously reported that the cut-off value of APRI score as 0.77 and 0.78 in European patients can predict significant fibrosis and cirrhosis, respectively [11, 12]. Moreover, multiple reports also tend to support that the APRI facilitates for us the prediction of the absence of both significant fibrosis and cirrhosis with negative predictive values over 90% [11, 12].

It is worthy of our attention that only few patients with S4 fibrosis and G4 inflammation were enrolled in this study, and patients with S3 fibrosis and G3 inflammation also represented a minor fraction of our study population. This distribution of disease severity reflects the fact that severe necroinflammation and cirrhosis are rarely detected in adolescent patients. However, this population cannot remove HBV infection and it is known that persistent viral infection can lead to persistent occult development of liver diseases and the occurrence of irreversible cirrhosis. Thus, we think the diagnostic usefulness of the APRI to indicate significant fibrosis (≥S2) or inflammation (≥G2) is important to identify disease progression in this young patient population, thereby facilitating timely treatment. A diagnostic tool is considered as a good tool if the AUROC is greater than 80%, excellent if the AUROC is greater than 90%, and perfect if the AUROC is 100%. According to these results, the APRI may not be considered as a good tool for predicting significant fibrosis and cirrhosis in hepatitis B-related fibrosis. However, APRI is a simple and cheap method and is suitable to monitor the dynamics status of some liver diseases [3, 4] and it can be performed as an auxiliary method of liver biopsy [14–18].

Conclusively, this study supported that the APRI model can predict significant liver fibrosis and (the cut-off point for prediction of significant liver fibrosis was 0.83) and inflammation (the cut-off point for prediction of significant liver inflammation was 0.85). The clinical significance of this index predictive of liver fibrosis and inflammation seemed advantageous in CHB patients with age of 6–12 y compared with age of ≤6 y or ≥12 y. Our data suggested that this index should be an important tool for auxiliary diagnosis. However, clinical significance of APRI in adolescent HBeAg-negative patients is still to be demonstrated.

## Supplementary Material

Answer: Table S1: Univariate analysis of variables associated with significant liver fibrosis in 88 adolescent patients with CHB. In this table, the clinical indicators including ALB, PLT, gender, age, total bilirubin (TBIL), APRI, Prothrombin time activity(PTA),ALB, Log10(HBV DNA), cholinesterase (CHE) were investigated with univariate analysis between 45 cases with mild liver fibrosis (S1) and 43 with significant liver fibrosis (≥ S2).
Table S2: Univariate analysis of variables associated with significant liver inflammation in 88 adolescent patients with CHB. In this table, the clinical indicators including ALB, PLT, gender, age, total bilirubin (TBIL), APRI, Prothrombin time activity(PTA), ALB, Log10(HBV DNA), cholinesterase (CHE) were investigated with univariate analysis between 31 patients with mild liver inflammation (G1) and 57 patients with significant liver inflammation (≥ G2).

## Figures and Tables

**Table 1:** The diagnostic value of the APRI for predicting significant liver fibrosis and liver inflammation.

Group	APRI value (cut-off)	AUROC (95% CI)	Sensitivity (%)	Specificity (%)	PPV (%)	NPV (%)	Accuracy rate (%)
Liver fibrosis stage ≥S2	0.83	0.73 (0.62–0.83)	76.7	71.1	71.7	76.2	73.9
	0.5	0.73 (0.62–0.83)	90.7	31.1	55.7	77.8	60.2
	1.5	0.73 (0.62–0.83)	27.9	88.9	70.6	56.3	59.1

Liver inflammation stage ≥G2	0.85	0.74 (0.63–0.84)	64.9	74.2	82.2	53.5	68.2
	0.5	0.74 (0.63–0.84)	87.7	35.5	71.4	61.1	69.3
	1.5	0.74 (0.63–0.84)	29.8	96.8	94.1	42.3	52.3

**Table 2:** The predictive value of the APRI for significant fibrosis (≥S2; cut-off = 0.83).

Age group	AUROC (95% CI)	Sensitivity (%)	Specificity (%)	PPV (%)	NPV (%)	Accuracy rate (%)
≤6 y	0.66 (0.48–0.84)	68.4	70.6	72.2	66.7	69.4
6–12 y	0.84 (0.68–1.0)	85.7	86.7	85.7	86.6	86.2
≥12 y	0.66 (0.43–0.88)	80	53.8	57.1	77.8	65.2

**Table 3:** The predicative value of APRI for significant inflammation (≥G2; cut-off = 0.85).

Age group	AUROC (95% CI)	Sensitivity (%)	Specificity (%)	PPV (%)	NPV (%)	Accuracy rate (%)
≤6 y	0.68 (0.49–0.87)	82.6	53.8	76	63.6	72.2
6–12 y	0.79 (0.62–0.96)	70.6	83.3	85.7	66.7	75.9
≥12 y	0.66 (0.43–0.88)	80	53.8	57.1	77.8	65.2
