# Supplementary material for: Role of the Aspartate Transaminase and Platelet Ratio Index in Assessing Hepatic Fibrosis and Liver Inflammation in Adolescent Patients with HBeAg-Positive Chronic Hepatitis B
Source: Gastroenterol Res Pract. 2015 Jul 5;2015:906026. doi: 10.1155/2015/906026 (PMC4506824; doi:10.1155/2015/906026)
Supplement: Supplementary file 1 — Answer: Table S1: Univariate analysis of variables associated with significant liver fibrosis in 88 adolescent patients with CHB. In this table, the clinical indicators including ALB, PLT, gender, age, total bilirubin (TBIL), APRI, Prothrombin time activity(PTA),ALB, Log10(HBV DNA), cholinesterase (CHE) were investigated with univariate analysis between 45 cases with mild liver fibrosis (S1) and 43 with significant liver fibrosis (≥ S2). Table S2: Univariate analysis of variables associated with significant liver inflammation in 88 adolescent patients with CHB. In this table, the clinical indicators including ALB, PLT, gender, age, total bilirubin (TBIL), APRI, Prothrombin time activity(PTA), ALB, Log10(HBV DNA), cholinesterase (CHE) were investigated with univariate analysis between 31 patients with mild liver inflammation (G1) and 57 patients with significant liver inflammation (≥ G2). [file 906026.f1.pdf]

Table S1: Univariate analysis of variables associated with significant liver fibrosis in 88 adolescent patients with CHB

Index	Patients with mild liver fibrosis (n=45)	Patients with significant liver fibrosis (n=43)	Univariate analysis P value
Age (years)	8.97±5.37	8.99±5.93	0.985
Gender male	32(71.1%)	25(58.1%)	0.203
female	13(28.9%)	18(41.9%)	
ALT(IU/L)	122.40±153.57	187.88±192.95	0.081
AST(IU/L)	83.71±61.60	130.26±109.04	0.017
AST/ALT	0.97±0.53	0.86±0.48	0.295
TBIL(umol/L)	9.68±5.30	9.40±4.70	0.791
CHE*(U/L)	7572.13±1281.39	7074.70±1289.61	0.073
PLT(×109/L)	255.38±70.51	227.28±57.74	0.044
PTA* (%)	101±17	93±13	0.021
ALB (g/U)	42.25±4.8	40.53±2.37	0.036
Log10(HBV DNA)	7.83±0.88	7.48±1.08	0.102
APRI	0.86±0.58	1.39±0.95	0.002

Note: PTA: Prothrombin time activity; CHE: cholinesterase.

Table S2: Univariate analysis of variables associated with significant liver inflammation in 88 adolescent patients with CHB

Index	Patients with mild liver inflammation (n=31)	Patients with significant liver inflammation (n=57)	Univariate analysis P value
Age (years)	7.98 ± 4.42	9.14 ± 5.28	0.299
Sex male	24(77.4%)	33(57.9%)	0.067
female	7(22.6%)	24(42.1%)	
ALT(IU/L)	95.61 ± 83.87	186.37 ± 203.50	0.004
AST(IU/L)	73.26 ± 37.06	124.51 ± 105.23	0.001
AST/ALT	1.01 ± 0.53	0.87 ± 0.49	0.217
TBIL(umol/L)	9.50 ± 4.79	9.63 ± 5.41	0.908
CHE(U/L)	8073.74 ± 1262.96	6924.07 ± 1142.87	0.000
PLT($\times 10^9$/L)	263.87 ± 70.17	229.75 ± 60.71	0.019
PTA (%)	100 ± 15	95 ± 15	0.189
ALB(g/U)	42.75 ± 5.04	40.68 ± 2.89	0.041
Log_{10}(HBV DNA)	7.87 ± 0.90	7.55 ± 1.03	0.145
APRI	0.75 ± 0.46	1.32 ± 0.90	0.000
